# Beyond the numbers of maternal near-miss in Rwanda – a qualitative study on women’s perspectives on access and experiences of care in early and late stage of pregnancy

**DOI:** 10.1186/s12884-016-1051-4

**Published:** 2016-09-02

**Authors:** Jessica Påfs, Aimable Musafili, Pauline Binder-Finnema, Marie Klingberg-Allvin, Stephen Rulisa, Birgitta Essén

**Affiliations:** 1 Department of Women’s and Children’s Health, International Maternal and Child Health (IMCH), Akademiska Sjukhuset, Uppsala University, SE-751 85 Uppsala, Sweden; 2 Department of Pediatrics and Child Health, College of Medicine and Health Sciences, School of Medicine, University of Rwanda, P.O. Box 217 Butare, Huye Rwanda; 3 School of Education, Health and Social Studies, Dalarna University, SE-791 88 Falun, Sweden; 4 Department of Obstetrics & Gynecology, College of Medicine and Health Sciences, School of Medicine, University of Rwanda, P.O.Box 3286, Kigali, Rwanda; 5 Department of Clinical Research, University Teaching Hospital of Kigali, BP 655 Kigali, Rwanda

## Abstract

**Background:**

Rwanda has made remarkable progress in decreasing the number of maternal deaths, yet women still face morbidities and mortalities during pregnancy. We explored care-seeking and experiences of maternity care among women who suffered a near-miss event during either the early or late stage of pregnancy, and identified potential health system limitations or barriers to maternal survival in this setting.

**Methods:**

A framework of Naturalistic Inquiry guided the study design and analysis, and the ‘three delays’ model facilitated data sorting. Participants included 47 women, who were interviewed at three hospitals in Kigali, and 14 of these were revisited in their homes, from March 2013 to April 2014.

**Results:**

The women confronted various care-seeking barriers depending on whether the pregnancy was wanted, the gestational age, insurance coverage, and marital status. Poor communication between the women and healthcare providers seemed to result in inadequate or inappropriate treatment, leading some to seek either traditional medicine or care repeatedly at biomedical facilities.

**Conclusion:**

Improved service provision routines, information, and amendments to the insurance system are suggested to enhance prompt care-seeking. Additionally, we strongly recommend a health system that considers the needs of all pregnant women, especially those facing unintended pregnancies or complications in the early stages of pregnancy.

## Background

Despite decades of global efforts focusing on decreasing maternal deaths, 800 women still die each day with approximately two-thirds of these deaths in sub-Saharan Africa alone [1, 2]. Restrictive abortion laws significantly increases risks of morbidities and mortalities, and an estimated 18 % of all maternal deaths in eastern Africa are due to unsafe abortions [3, 4]. Therefore, the post-2015 maternal strategy of ending all preventable maternal mortalities is based on a holistic and rights-based approach, with focus on inequities to access, quality, and outcome of care, and provision of information and adequate options for all girls and women to exercise their own reproductive choices [2].

Over the past decade, Rwanda has strengthened its health system and maternity services. Maternal mortality ratio has decreased from 487 in 2010 to 210 in 2015, and facility-based deliveries have increased from 69 % in 2010 to 91 % in 2015 [5, 6]. Reasons for this increase may be due to the rule that imposes a fine if women deliver at home, the outlawing of traditional birth attendants or the availability of health insurance [7, 8]. Women’s financial situation and health insurance coverage play a key role in facilitating their care-seeking [9, 10]. The community-based health insurance, “Mutuelle de Santé,” (herein, Mutuelles), allows members to pay only 10 % of the care costs and some medicines [11, 12]. Currently 73 % of the population is enrolled [13]. Civil servants and military, with respective family members are covered by their own insurance scheme [14].

Although modern contraceptive use has increased tremendously in Rwanda, nearly every second pregnancy is unintended, and 22 % are estimated to result in induced abortions [5, 15]. Rwanda revised its abortion law in 2012 to allow abortion for pregnancies resulting from rape, incest, forced marriage, or if the pregnancy poses a health risk to the woman or fetus [16]. However, to be allowed a legal abortion, approval from the court and consent from two medical doctors is needed. Given this laborious approval procedure, and the cultural and religious stigma associated with abortion in this setting, abortion continues to be difficult to obtain [16, 17]. Notably, despite having complications requiring treatment, one-third of women do not seek post-abortion care [15]. A recent hospital-based study on near-miss during pregnancy in Kigali showed that 45 % of all severe morbidities and 28 % of mortalities were abortion related [18].

The obstetric phenomenon of near-miss is defined as “a woman who nearly died but survived a complication that occurred during pregnancy, childbirth, or within 42 days of termination of pregnancy” [19]. WHO has introduced certain clinical, laboratory, and management-based criteria to define near-miss in a standard manner [20], which have been further modified for low-resource settings [21]. The near-miss approach functions as a quality indicator of maternal healthcare and a proxy for maternal mortality [19]. These women often share similar trajectories with women who do die, but they can give their perspective of what actually happened, which helps to facilitate the understanding about their barriers to accessing maternity care [22, 23]. Relatively few studies have included near-miss events in early pregnancy, an aspect often overlooked in the maternal health dialogue, and even fewer have looked beyond the numbers [18, 24, 25].

Our aim is to explore care-seeking and experiences of maternity care among women who suffered a near-miss event in the early or late stage of pregnancy, and to identify potential barriers and health system limitations to maternal survival in this setting.

## Methods

### Study setting

Kigali provides public and private health centers, as well as three district and three tertiary referral hospitals, which serve an urban and peri-urban population of 1.2 million. The health system has a pyramidal structure. At the grass-roots level, the community health workers (CHW) inform and encourage pregnant women to seek maternity services, and accompany for labor if needed. When in labor, a woman first turns to a community health center and is referred to higher-level district or referral hospital, if required [7]. Post-abortion care is available in private and public health centers and hospitals [15]. Participants in this study were sampled purposively at three of the referral and district hospitals to be able to capture women from Kigali and referred cases, between March 2013 and April 2014.

### Inclusion criteria and definitions

Inclusion in the study was decided by the near-miss criteria, which was defined by local obstetricians in line with criteria used earlier in other low-resource contexts [21, 22]. This included: shock, emergency hysterectomy, uterine rupture, sepsis or signs of severe infection (temperature >40), the hypertensive diseases eclampsia and severe preeclampsia, management-based criteria of blood transfusion (adapted to ≥3 units of blood), and severe anemia (<6 HB). In this paper, we define “early stage of pregnancy” as the period within 28th week of gestation, and “late stage of pregnancy” as any time point after the completion of the 28th week of gestation. The limit of 28 weeks of gestation was chosen because this is according to the cut off gestational age for fetal viability in this setting.

### Data collection

Ethical approval was obtained from the Rwanda National Health Research Committee, Kigali (NHRC/2012/PROT/0045) and verbal informed consent was obtained from all study participants. The near-miss cases were identified at each hospital by the research team in collaboration with local obstetricians, midwives and nurses. The team approached each identified woman, explained the study objectives and asked whether she wanted to participate. The women were informed about confidentiality, future data storage, and their right to withdraw from the study at any given time without reason. When eligible participants were deemed physically healthy and gave informed consent, they were interviewed in their hospital room or in a private room. The interviews occurred within the first week following the near-miss event. The women were contacted for a follow-up session within nine months after the initial interview if they lived within two hours of Kigali and had given consent for a follow-up. The participants were interviewed either in their home or in a restaurant having a private room.

A framework of Naturalistic Inquiry guided the study and its dialogical approach was used for the interviews, which resembled a conversation more than a question-answer interview, using open-ended questions [26]. The themes covered during the interviews were participants’ care-seeking, perceptions on access and experiences of care during pregnancy, the near-miss event, and future reproductive health desires. Prior to the study, four pilot interviews were done to validate the preliminary interview questions, additional questions emerged throughout the study as is typical for the chosen method [26]. The first author and a female local translator conducted all interviews, and received training in interview techniques by the third author, skilled in qualitative research. To verify our interpretations of the findings, we did member checks throughout the study period with the informants and community members, as well as to help define emergent interview questions [27].

Participants included 47 women for first-round interviews and 14 of these for follow-up. All interviews, except one, were digitally recorded. Each first-round interview took 20–60 min, and follow-up interviews 1–2 h. The interviews were conducted in Kinyarwanda and translated into English with the help of two interpreters. Eighty percent of the interviews were cross-checked and validated for consistency by additional interpreters, who were unknown to each other.

### Analysis

Early analysis commenced during fieldwork by coding and summarizing women’s narratives and field notes. When all interviews were completed, the first author re-read all transcripts and field notes, and recoded them, in discussion with the co-authors. To conceptualize barriers to seeking and receiving care in this setting, data were sorted with inspiration from Thaddeus and Maine’s (1994) ‘three delays’ model: Phase I: decision to seek care; Phase II: identifying and reaching medical facility; Phase III: receiving adequate and appropriate treatment [28]. We created a matrix with separate columns for early and late stages of pregnancy, and color-codes for marital status, for whether the severe obstetric complications arose prior or at arrival to the facility, and if care had been sought repeatedly. Separating near-miss events upon and after arrival helped to facilitate the distinction between potential pre-facility barriers and quality of care [24, 29]. We compared the overall similarities and differences of women’s narratives and field observations, and noted resemblances and contrasts in trajectories and experiences of care.

### Results

Most women lived in the peri-urban communities of Kigali. Age, educational level, and parity (Table 1) varied among the participants. The marital status also varied, and some had been abandoned by their partners because of the pregnancy or the near-miss event itself. All except five women belonged to the lower socioeconomic group, according to their occupation and whether they were targeted by the community based health insurance, Mutuelles.

**Table 1:** Characteristics of the women and their near-miss events, separated according to week of gestation

Near-miss cases	Early: ≤28 weeks (*n* = 21)	Late: >28 weeks (*n* = 26)
Upon arrival	20	5
After arrival	2	22
Defined as repeated care-seeking attempts	10	4
Diagnosis^a^		
Hemorrhage	10	15
Hypertensive disorder	1	9
Infection	8	6
Obstructed labor	0	1
Anemia	3	0
Age (years)		
< 20	1	1
20-24	7	8
25-29	6	4
30 <	7	13
Marital status		
Single/Separated	10	3
Married/Cohabiting	11	23
Education		
Primary	13	15
Secondary	5	6
Upper level	1	3
None or unknown	2	2
Community-Based Health Insurance		
Yes	13	21
No	8	3
Other insurance	0	2

^a^Six women had two near-miss events as defined by a physician, midwife or nurse

The women arrived either at the facility in a life-threatening condition or had developed the complications at the facility, or both. A few had been identified as “high risk” cases either by a healthcare provider or by a CHW, and been admitted prior to labor. In most cases, however, the women reported that they either had no trust in the person assigned as CHW, or that the person had not been active during their pregnancies. Among the women with near-miss in early pregnancy, all pregnancies terminated before gestational week 28, and no fetuses survived. Among women in late pregnancy, eight were either stillborn or did not survive during the neonatal period.

**Fig. 1:**
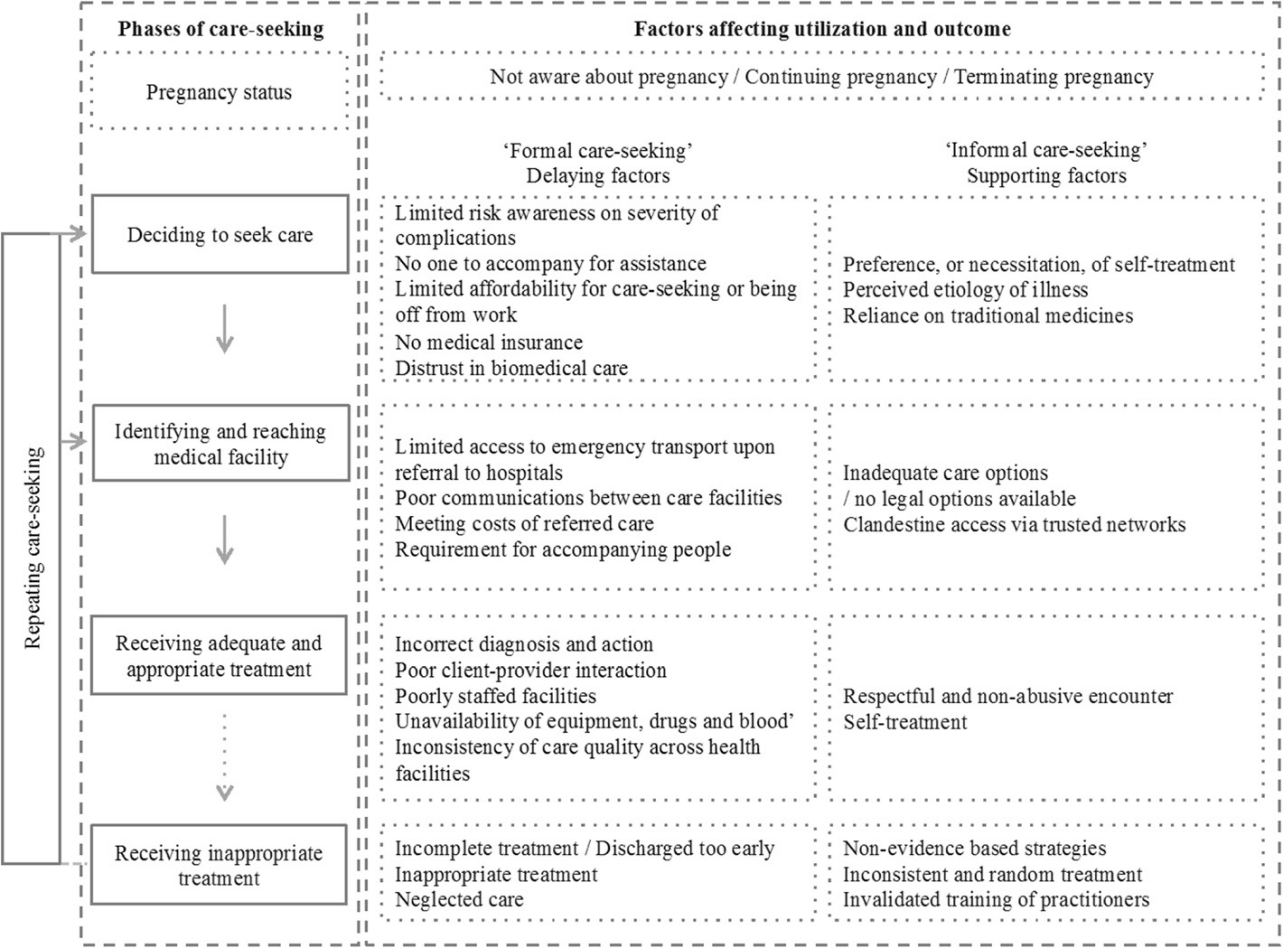
Presenting the different phases of care-seeking, clarifying the “formal” and “informal” routes

Factors that delayed or supported care-seeking are shown in Fig. 1. Care sought “formally” refers to private and/or public healthcare facilities providing maternity services within the formal health system. “Informally” refers to solutions sought outside the defined health system. Four main themes were identified: *(i) Pregnancy status, (ii) Barriers in seeking and reaching appropriate care, (iii) Inadequate counseling and repeated care-seeking, and (iv) Continued adherence to traditional medicine*.

### Pregnancy status

Unmet need for contraception and unintended pregnancies were common themes in women’s narratives and appeared to influence the decisions women made about their pregnancy status. For some, the modern contraceptives had either failed or the women had decided to stop because of side effects. Others had relied on traditional methods of counting, been breastfeeding or unaware of the potential to conceive. Some had attempted to use condoms but had failed, or described partner refusal. The younger women relied on either condoms or counting, or nothing, and had low awareness of modern long-lasting methods.

Only one of the single women described her pregnancy as intended and wanted, while the rest expressed them as unwanted. This varied among women in a relationship, and several had not been happy about the news. One woman of five children said she “came to terms with it,” yet described ambivalence towards it:

I never considered having an abortion. But when it was four months, when I used to see some small bleeding, I thought about it and I wished it could go away. But I never thought of doing it myself. (#43: 36 year-old, grandmultipara, late pregnancy).

Some of the women had intentionally terminated their pregnancy based on their desire to not become a mother, inability to financially provide for a baby or lack of support, or the expectant father’s desire to not keep the pregnancy. In some cases, the expectant father was absent altogether. The decision to abort had been taken in isolation, or after seeking advice from a trusted friend or relative. The women had turned either to a traditional healer or clinic offering this service clandestinely. Some attempted to terminate the pregnancy themselves. The latter had occured among the women who were unable to pay for those services, or were unaware of how to access them. These women aborted by either eating several contraceptive pills, drinking glycerin or other “mixtures,” or by using cassava sticks, which were thought to contain a toxin to provoke an abortion. One single woman explained:

I got two cassava sticks, went to the toilet and inserted them deep into my vagina. I hated myself in that moment, but I was reasoning that since my parents are dead, and I don’t have a husband, I would not have been able to take good care of that baby. So, I felt like the best option was to abort that pregnancy (#6: 29 year-old, nullipara, early pregnancy).

Another single woman explained how she “went to a place to take care of it, but somehow they failed” (#16: 24 year-old, nullipara, early pregnancy). For one of the women, the abortion was triggered by physical abuse from her partner: “He was not happy about the pregnancy […] When I was three and a half months pregnant, he punched me from the back and I started bleeding” (#17: 38 year-old, primipara, early pregnancy). A few of the women with early-pregnancy near-miss explained the termination of pregnancy as unexpected, with some unaware of being pregnant. One particular case was a 16 years old girl with near-miss in late pregnancy who also expressed the termination of pregnancy as unexpected. She had not disclosed her pregnancy to even her mother because: “I feared that she would beat me up”. In the eighth month of pregnancy, her mother took her to a traditional healer for something they believed was an intestinal worm. The girl described how she had a miscarriage on the toilet when she came home: “I started to bleed a lot. One neighbor found the baby and called the police” (#26: 16 year-old, nullipara, late pregnancy). The woman was being investigated by the police, and suspected for voluntary abortion. A few others also reported being accused for voluntary abortion, and subsequently, because of it, were left by their intimate partner or lost their jobs.

### Barriers to care-seeking and reaching appropriate care

The decision to seek care was influenced by the perceived need for treatment and quality of care. Symptoms throughout pregnancy, such as swollen feet, bleeding, pain or not feeling fetal movement, were seldom referred to as reasons to seek care. The women relied on, and sought advice from, their closest social network of family, friends, neighbors, CHWs, or traditional healers. However, among those with symptoms in early pregnancy, hesitation in both consulting others and seeking care were evident and described in the women’s narratives about having thought or wished for the symptoms, mostly bleedings, to stop by itself. The shameful and stigmatizing label of being pregnant outside a partnership was a contributing reason, and these pregnancies were often kept secret. Among the single women who had intentionally terminated their pregnancy, they had tried to hide the pain and bleeding. In each of these cases, the woman was found in a critical condition by a family member or neighbor, and brought to the facility for care. In contrast, married women appeared to postpone seeking care less when symptoms arose, partly because they felt protected from the suspicions and stigma of self-terminating a pregnancy: “Since I am a married woman, people did not seem to care. It is unthinkable that anyone could provoke an abortion if you are married” (#2:27 year-old, multipara, early pregnancy). However, if the pregnancy outcome was negative, some women expressed barriers to reaching appropriate care. Despite heavy bleeding, one participant described being denied care at a private health center: “When I got in the doctor said he wasn’t going to do anything for me without my [partner] also being there […] he said he can’t do anything because they always shut down his dispensary” (#12:24 year-old, multipara, early pregnancy). Women perceived this precautionary measure as the healthcare provider trying to avoid accusations about having performed an abortion.

Limited affordability and/or not being covered by Mutuelles were presented as an important factor of delay in both early and late pregnancy. The ability to pay while seeking care was perceived as important, particularly because: “if you don’t have money to pay they lock you in at [the health facility] for a long time” (#22:30 year-old, primipara, late pregnancy). Unemployment, shortage of money and/or lack of support from the partner or other relative had delayed care-seeking for several participants. In addition, being covered by Mutuelles was perceived as a necessity to be able to manage costs. However, a delaying factor for enrolling in the health insurance program was that everyone in the family had to sign-up and pay immediately upon enrollment. Obtaining the insurance was also perceived to sometimes take a month or longer. Several women had enrolled only when they learned they were pregnant, or according to the perceived due date, which was sometimes wrongly estimated by healthcare providers. Paradoxically, the reliance on the due date also caused some women to delay their care-seeking. This appeared to be caused by an obedience to guidelines and fear of coming ‘too early’ to the health facility, which was perceived to not be appreciated by healthcare providers. There were a few cases where the women had waited with seeking care, as they believed their symptoms were not labor related. For example: “I thought I was not supposed to go to the hospital yet as it was still not the due date they had provided me with. Therefore I delayed” (#30:21 year-old, primipara, late pregnancy).

The concept of witchcraft was described and understood as a reason for potential complications during pregnancy. Witchcraft was referred to as someone wanting to harm the woman or her pregnancy because of jealousy or hatred towards her or her family. These perceptions also contributed to delayed care-seeking, either because women did not want to disclose their pregnancy before it was visible, or because biomedical care was not considered applicable. For example: “I thought I’d been bewitched, so I didn’t go directly to hospital. A lot of people had told me it was possible to have this kind of bleeding if a person is bewitched” (#8:30 year-old, multipara, early pregnancy).

Distrust in care was prominent, and being accompanied by someone was perceived as important for facilitating admission, as well as organizing needed supplies and providing food while at hospital. Problems with reaching the actual care-facility were not noted, but flaws in the referral chain were pointed out in some narratives and appeared to be due to lack of transport or because of constraints in the communication between and within facilities. In a few cases, the receiving hospital had been unprepared for an emergency case. One woman explained how she was still lying in the ambulance and could “hear [the nurse] arguing that another hospital was responsible for the referral” (#47: 23 year-old, primipara, late pregnancy). She was finally admitted after what she described as a moment of stress.

### Inadequate counseling and repeated care-seeking

Missing supplies, lack of staff, poor patient-provider interaction and suboptimal treatment, as described in the women’s narratives, were identified as main barriers to an optimal care encounter. Repeated care-seeking occurred because women had either been misdiagnosed, received incomplete care, discharged too early, or received inappropriate treatment altogether. These were identified among women both in the early and late stages of pregnancy, and appeared to have contributed to some of the near-miss events.

Among the women who had near-miss in early pregnancy, a few had received contraceptives, or another form of treatment without knowing they were already pregnant. When the symptoms occurred, such as bleeding, some described being unaware of any danger and had therefore delayed with seeking care. Others described that they sought care but were advised to return home. One woman who sought care directly after her miscarriage explained: “I returned home because the nurse told me that since I had aborted I didn’t need to go to the hospital. I went home, buried the baby and stayed home for about a week” (#21:24 year-old, nulliparous, early pregnancy). This woman’s condition worsened, and when she finally returned to the health center, she was in sepsis and immediately referred to the hospital where one of her fallopian tubes was promptly removed. Another woman was discharged after being treated with three blood transfusions due to post-abortion bleeding. We visited her at home a few days after and found her with a high fever. The inpatient services during her near-miss event had put her in debt and she was not planning to return for care before obtaining the insurance. She said her application was in progress and she waited for a decision. She stayed home for some additional days and when her condition worsened, she was again brought to a health center, from which she was immediately referred to the hospital because of sepsis caused by a retained placenta, and she experienced a second near-miss event.

Most women with a near-miss in late pregnancy had attended one antenatal check-up, but only a few had attended more than once. Several reported that healthcare providers had not been engaged in dealing with their symptoms or health concerns. Retrospectively, they believed their problems could have been avoided had the providers paid more attention and informed them of potential complications. This was especially apparent among women with hypertensive disorder. Their early signs were missed in consultations, misdiagnosed or not taken seriously, even though women had repeatedly sought advice at the health center. One woman explained: “When I went to the health center for the antenatal check-up, they always told me I had no problem. They said I should not work so hard and gave me pain-killers whenever I complained of pain” (#15:24 year-old, primipara, late pregnancy). Some women described having limited trust in the advice or the medicine given to them. One woman sought care because of pain during early pregnancy and the healthcare provider wanted to treat her with what she recalled as an injection and pills. She refused: “I asked him how he could inject me and give me pills without consulting me to be sure about what I had […] I refused because I didn’t trust those medicines because I thought they could also be harmful to my pregnancy” (#12:25 year-old, multipara, early pregnancy). She later miscarried and bled heavily, leading to a transfusion when she arrived at the health facility.

Limited ability to pay, and Mutuelles not reimbursing the costs for all medicines appeared to be a contributing factor to the incomplete treatment. One woman explained how she had asked to be discharged earlier than necessary because she could not afford inpatient treatment. The participant who sought care due to a threatened abortion after physical abuse from her husband said: “[The doctor] prescribed me some medicines but they asked me to go and buy them on my own. When I got home, my husband refused to buy those medicines” (#17 38 year-old, primipara, early pregnancy). She miscarried, lost blood, sought care again and was diagnosed with severe anemia for which she was unable to afford the medicine. However, she further explained that the doctor had bought her the medicine when he learned she was unable to afford it. Other women confirmed the struggle of affording medicine, particularly for treating anemia, as Mutuelles did not reimburse this medicine. Others faced challenges such as affording equipment or the expense of an emergency surgery. In some of these cases, the healthcare providers had helped out: “One of them bought me an injection, and another added the amount that was left from what my husband had so we could buy the medicine” (#31:22 year-old, primipara, late pregnancy).

Women understood giving birth at a care facility as obligatory, and most were supportive of this policy but questioned the fee system if they did not reach a facility on time. However, women raised complaints about being disrespected at the care encounter, as well as about how healthcare providers dismissed their obstetric history and their own expressed needs. In particular, women with earlier experiences of complicated births had actively tried to prevent future complications but felt overlooked. These women met such comments as: “Who do you think knows what to do here, us or you?” (#45:29 year-old, multipara, late pregnancy).

Overall, the underlying reasons for the near-miss outcome were rarely explained to the women. For some, their survival was paramount, and they had no interest in knowing the details of their diagnosis. One woman, who was informed about her hypertensive disorder, explained, “They told me I had a very serious disease that can kill. But, I don’t believe in that because I put my trust in God” (#23:25 year-old, primipara, late pregnancy). Several women wanted to know more, but expressed difficulties in obtaining an explanation:

“Every time I asked the nurse to tell me what was going on, they would tell me that it is only up to the doctor to tell me that. However, when the doctor comes to see you, he only asks how you are doing and doesn’t bother to tell you what is causing your problem” (#14:26 year-old, multipara, early pregnancy).

For some women, their complications arose post-treatment, mostly due to retained placentas or other infections developing into sepsis. These women had not received counseling on symptoms to pay attention to, and delayed seeking care again because they perceived the symptoms as part of the recovery process. One raised this complaint: “All of them, including those who were discharged at the same time as me, were told nothing” (#12: 25 year-old, multipara, early pregnancy). It was apparent from the follow-up interviews that only a few of the women had been offered contraceptive counseling before being discharged. At the time of the interview, several were not using any method of protection.

The overall limited exchange of information seemed to create an atmosphere of distrust among the women with the care that was provided to them, or deepened their own reasoning on the explanations for what happened. One woman with a stillbirth, whose hypertensive disorder had gone undetected, had not received any explanation from the healthcare provider, and reasoned: “I have a belief that there is one lady who bewitched my baby inside the womb, because, I used to feel my baby moving, with no problem, but after that lady visited me, my baby died” (#42:20 year-old, primipara, late pregnancy). For others, the discontent with care provided made them turn to a traditional healer. One participant had sought biomedical care repeatedly without receiving explanations about her persistent bleeding, and decided to turn to a traditional healer. The bleeding initially stopped but returned after some days and became severe. Her partner brought her to a public health center, and she was referred to the hospital, where it was discovered she had a ruptured ectopic pregnancy. Immediate actions were taken: “They decided to operate me as an emergency, even before other people they had been scheduled to operate” (#20:30 year-old, multipara, early pregnancy).

### Continued adherence to traditional medicine

Adherence to the use of traditional medicine was prominent in women’s care-seeking during pregnancy, particularly right before birth. Traditional medicine was taken as a preventive measure, either orally or applied on the belly, a process called *kwitegura*, which translates as “to get ready”. The “medicine” was taken in secret and seldom revealed to the healthcare providers because the women perceived that most biomedical health facilities would impose a fine if they found out.

One reason for turning to a traditional healer before giving birth was described as precautionary measure and explained as, “If a woman has been bewitched she won’t die while delivering” (#47:23 year-old, primipara, late pregnancy). Besides the protection from potential witchcraft, the medicines were also described as a protective agent for both the mother and child from certain sicknesses or for helping to facilitate childbirth, mostly by increasing the contractions. Another aspect was distrust in the quality of available biomedical care, as one woman described:

I was due but my contractions had not started and I knew that if you go to [the clinic] without contractions, they are not going to help you. My husband bought a small dose of traditional medicine made from herbs. It helped me. If I hadn’t taken that medicine, I wouldn’t have given birth (#36:37 year-old, multipara).

Cost was the final contributing factor described by participants that helped them turn toward traditional medicine. The women described having the option to delay paying for traditional medicines, or to pay for them in installments, neither of which were offered by the formal facilities. In addition, whereas sometimes the medicines were used willingly, many described use of informal treatments as having been imposed by a family member, mostly the mother, mother-in-law, grandmother or sister. Use of traditional medicines for pregnancy and childbirth was confirmed as common practice, particularly in certain areas of Kigali and the rural context. A few of the participants, however, stated they had not taken these measures because it conflicted with their religious beliefs. Others perceived them as harmful or not needed in their urban community.

## Discussion

This study identified various trajectories to near-miss events. Women confronted different barriers depending on whether the pregnancy was wanted, the gestational age, their insurance coverage, and their marital status. The latter was particularly important, and is an indicator to access and receipt of appropriate care in early pregnancy [30]. Women’s unmet need for contraceptives was prominent, highlighting a necessity for improved access to reproductive health information and services. It is suggested that one-third of maternal deaths could be averted if the need for contraception is met; however, this will not eliminate unwanted pregnancies completely [31, 32]. The women wanting an abortion faced an absence of care options and put their own lives at risk in the process. It is nonetheless probable that “safe” options are available, especially in the urban setting, by clandestinely paying someone to discretely perform them. Yet, to access these options, one must have reliable networks and financial resources, which are often not available to women in low socioeconomic groups. These women are thus forced to take other less desirable measures [15]. These findings highlight important aspects of inequity in this setting.

Given the stigma and criminalized status of abortions, women with early-pregnancy symptoms were simultaneously cautious about seeking care and sometimes suspected of being responsible for the near-miss event, especially if single. Notably, one of the single women who had a late miscarriage at home was reported to the police by a neighbor. In Rwanda, women imprisoned for abortion are often reported by someone from their closest social network [17], which may serve to increase community distrust. Rwanda may be exceptional in its reporting system and implementation of the abortion law, highlighting the question of gender inequity, as it is the woman who is sentenced to prison, but not the partner, who might have organized it or potentially forced her to terminate the pregnancy [33]. The Rwandan Penal Code, Article 163, states that a person who causes a woman to abort is liable to imprisonment [16]. Whether this law is realized, in practice, warrants further exploration. Another aspect of gender inequity is the presence of social sanctions imposed on women who are suspected of aborting, such as losing a job or their reputation, and lastly, being denied appropriate care. In agreement with our findings, women seeking post-abortion care are likely to be deprioritized, or even denied care altogether [34]. In addition, it may be that single women are more prone to face discrimination. Our findings suggest that married women perceived they experienced less suspicion of having conducted an abortion. Nevertheless, one woman’s partner was still asked by the physician to be present for her to gain access to care. The criminalized label of abortions may pose inner moral conflicts, or fear of being suspected as responsible, and can also inhibit healthcare providers from performing their job, as suggested by a recent systematic review [35]. This is a subject for further research in Rwanda.

Due to inappropriate treatment, some of our participants had sought care repeatedly. This calls for an improvement in the service provision routine. Hypertensive diseases left unnoticed or undiagnosed strongly suggests a need for improved education about warning signs, as well as other obstetric complications, among providers, the community, and childbearing women. Although Rwanda has an increasing rate of facility-based deliveries, this does not support that all healthcare providers are skilled or that morbidities and mortalities have decreased accordingly [18]. Within the ambition to reduce adverse maternal outcomes, our findings highlight that healthcare providers must think beyond biomedical qualifications, and work toward improving communication to make services appealing and trustworthy to women and their social networks [36]. Women’s perceptions about being overlooked seems to have contributed to their reluctance to raise concerns – or to adhere to biomedical care altogether – which is also explained as a reason for the poor uptake of antenatal care in this setting [37]. Our findings echo the long-established discourse of treating women of lower socioeconomic and educational levels as unable to comprehend, and therefore not provided adequate explanations [38, 39]. This is in contrast to women of higher socioeconomic and educational levels, who usually dare to demand explanations [40]. Our findings raise awareness about the importance of respectful care services and provision of information to all women, particularly as women consult and trust each other.

Being pushed to seek care repeatedly may reinforce existing inequities since women from lower socioeconomic groups are considered to be more affected [41]. We found it interesting, that to balance the current constraints of the health system, some healthcare providers sometimes assisted by paying for the women’s services themselves. This act of kindness is appreciated, but may lead to discrimination if only certain types of patients are randomly helped. In addition, despite being insured, the women still faced difficulties, especially if unexpected surgery or unsubsidized medicines were needed. Thus, amendments to the insurance system for improvement of universal health coverage are needed. Importantly, near-miss women are more likely to face catastrophic and unanticipated health expenditures, as well as severe health consequences that make them unable to return to income generating activities [41, 42]. As our findings suggest, the loss of employment due to social repercussions is worthy of attention. We thus want to emphasize the need for a *continuum of care*, both preventive and post-treatment, including contraceptive counseling to prevent unintended pregnancies.

Medical pluralism and the social interpretation of illness are not new phenomena during pregnancy, but are often overlooked in the global and local promotion of biomedical strategies [43]. For instance, one indicator of the fifth millennium development goal (MDG5) was to increase the frequency of births attended by skilled personnel [44]. This might have contributed to the ban on homebirths and the usage of traditional medicine, causing conflict with local reasoning [45, 46]. Change remains crucial as usage of traditional medicine during pregnancy is suggested to contribute to morbidities and neonatal death, or can delay or dissuade women from seeking biomedical care altogether [45, 46]. Pregnant women will continue to utilize traditional medicines if an illness is perceived to be a result of witchcraft [38, 47]. Our findings further highlight a paradox in the value of traditional medicine; women expressed concern over being penalized for violating guidelines; however this did not seem to deter women from using it. Recommended ahead of penalizations are incentives that inform, meet, and challenge the sociocultural beliefs and practices of traditional medicines in a respectful manner.

### Strengths and limitations

This paper explores women’s narratives about maternal near-miss among those who reached the hospital on time. A potential limitation is the lack of inclusion of women who never made it to the health facility. We interviewed the women while still at the health facility to help facilitate immediate recall of the event. However, this milieu and the criminalization of induced abortions have likely contributed to some women being less outspoken. The necessity for building trust between the researchers and women was relevant, and we employed follow-up interviews to help provide consistency and to show the women our enduring interest in their experiences. The interviews conducted outside of the facility provided a more nuanced picture of the care encounter, suggesting women will be more reluctant to report criticism about care if interviewed at the hospital [23]. The first author was dependent on language interpretation, which was a reason for having more than one external interpreter double-check the transcripts for mistranslations.

## Conclusions

This study contributes to an improved understanding of the chain of events leading to increased risk for severe maternal outcomes in Rwanda. Inequities in exercising one’s own reproductive choices are highlighted. Although Rwanda presents a high number of women delivering at health facilities, adherence to preventive measures outside of the formal health system continues. Due to inappropriate treatment, women are pushed into repeated care-seeking, emphasizing the need for improved communication and continuum of care, and amendments to the insurance system. Trust matters, and preventable maternal mortality will not be eliminated if women or their closest social network trust neither the biomedical care provided nor each other. Questions remain about how to build trust in a system that labels certain symptoms during pregnancy as criminal. To further decrease cases of maternal morbidity and mortality, the maternal health services need to meet women’s actual needs and be trustworthy for all.
